# Supplementary material for: Performance of doubled haploid maize (Zea mays L.) testcross hybrids under optimal and drought-stressed environments
Source: PLoS One. 2026 Jun 3;21(6):e0350861. doi: 10.1371/journal.pone.0350861 (PMC13232817; doi:10.1371/journal.pone.0350861)
Supplement: Attachment — Submitted filename: Response to Reviewers_Final.docx [file pone.0350861.s002.docx]

**Performance of Doubled Haploid Maize (Zea mays L.) Testcross Hybrids Under Optimal and Drought-Stressed Environments**

**Dear editor and reviewers,**

**Thank you very much for your critical and constructive comments on our manuscript. We found that all your comments, questions, and suggestions are relevant and meant to improve the quality of our report and maintain the standard of the journal. Thus, the manuscript revised thoroughly: a**ll the reviewers concerns were considered carefully and the suggestions were incorporated in the revised manuscript. **Please refer the table below for our responses line by line.**

|  | **Reviewer # 1** | |
| --- | --- | --- |
| **No** | **Comments/Suggestion/Questions** | **Authors Response** |
|  | **Abstract** |  |
| 1 | Clarify the specific heterotic groups (e.g., Group A vs. Group B) mentioned in the hypothesis to provide immediate context for maize breeders. | Addressed on page 2 line 34 - 38 |
| 2 | Explicitly state the selection pressure applied (e.g., top 1.1% of 855 hybrids) to highlight the rigor of identifying the ten best performers. | Addressed on page2 line 44-45. Over all the abstract part improved |
|  | **Introduction** |  |
| 3 | In the introduction, move beyond general drought statistics to specifically address the increasing frequency of mid-season "managed drought" scenarios common in Sub-Saharan Africa. | Page 2, line 59-62 |
| 4 | Briefly mention the time-saving advantage of Doubled Haploid technology compared to traditional inbreeding to underscore the study's relevance to "rapidly developing germplasm". | Improvement reflected o**n page3 line 78-82** |
|  | **Material and Method:** |  |
| 5 | Explain the "common checks" strategy used to link the 17 trial sets more clearly, ensuring the reader understands how 855 hybrids were compared fairly. | The common checks strategy used to link the 17 trials has been briefly explained on **Page 4, line 110-112** |
| 6 | Quantify "optimal" vs. "managed drought" in terms of millimeters of water applied or soil moisture depletion levels. | **Improved as follow:**  Drought stress was imposed by withholding irrigation approximately two weeks before flowering and maintaining water deficit conditions until harvest, allowing progressive soil moisture depletion. In contrast, well-watered trials received supplemental irrigation whenever moisture stress was observed, although the exact amount of water applied was not quantified **(page 4, line 115-120** |
| 7 | Ensure all software versions (DeltaGen, R-software 4.4.1) are consistently cited with their respective developers or URLs. | Citations were maintained as advised, **Page 5 line 150, and Page 6 line 177 182, and page 7 line 187** |
|  | **Result** |  |
| 8 | Explicitly discuss the 37.73% Coefficient of Variation (CV) for the Kiboko_DT trial; explain if this is typical for high-intensity drought stress in this region. | **The discussion is presented as follow:**  The coefficient of variation (CV) for grain yield was highest under the Kiboko moisture-stressed condition (37.73%) compared to the optimal environments, indicating substantially greater relative variability under drought stress (Table 2). This elevated CV is expected under high-intensity drought conditions (just before two weeks of flowering and onward), particularly in drought-prone environments such as Kiboko, where water limitation amplifies differences among genotypes. Under stress, small differences in drought tolerance mechanisms such as root architecture, stomatal regulation, water-use efficiency, problem of synchrony of male and female flowering which enforce limited fertility can lead to large differences in grain yield, thereby increasing variability relative to the mean. Furthermore, drought stress typically reduces the overall mean yield (2.38 t ha⁻¹ in this case), which inherently inflates the CV since it is expressed as a percentage of the mean. The combination of reduced mean performance and heterogeneous genotype responses to stress contributes to the observed high CV **(Page 7-8, line 213-223)** |
| 9 | Provide a summary sentence noting that heritability for phenological traits (AD, PH, EH) was generally higher than for grain yield (GY) across all environments. | **The summary is given as follow:**  The result showed that, under optimal management at Kiboko, the heritability estimates for phenological and plant architectural traits (AD, PH, and EH) were consistently higher than those for GY across environments, indicating greater genetic control and stability for these traits **(page 7, line 208 - 211).** |
| 10 | Explain the statistical weighting used for Pop 17 and 18, given they were represented by only two and four genotypes respectively. | Genotypes from pop17 and 18, each represented by very few genotypes (n = 2 and n = 4, respectively) clustered only under cluster 4, showing limited distribution across clusters (Table 12), which likely reflects their small sample size and reduced representation of within-population variability **(Page 23 line 514-516)**. |
| 11 | Clearly state which traits (e.g., GY, EPP) were the primary drivers of PC1 and PC2 variation based on their vector lengths in Figure 6. | Traits with longer vectors in Figure 6, particularly GY and key plant architectural traits, contributed most to genotype discrimination and were the primary drivers of phenotypic diversity. Specifically, GY exhibited a longer vector than EPP along both PC1 and PC2, indicating that GY has stronger influence on the variation explained by the first two principal components (**Page 27 line 578-581**) |
| 15 | Emphasize that only seven genotypes (5.5%) were common across all environments to reinforce the "strong genotype x environment interaction" finding. | Only seven genotypes (5.5%) were common across all environments further emphasizes the strong genotype × environment interaction and suggests limited stability of genotypic performance across diverse testing conditions **(page 17 line 392 - 394)** |
|  | **Discussion** |  |
| 16 | Compare your 161% yield advantage finding with other tropical DH studies to show if your genetic gains are standard or exceptional. | The top 10 hybrids showed strong grain yield performance compared with the commercial checks, with average superiority of 161% under drought and 60.4% under optimum conditions. When compared with previous tropical doubled haploid (DH) studies, these gains appear exceptionally high. For instance, DH-derived maize hybrids evaluated in East Africa typically showed yield advantages ranging from about 23% under optimum conditions to approximately 43% under drought stress relative to commercial checks (Sserumaga et al. 2018). Similarly, other tropical hybrid improvement studies have reported yield gains of approximately 21–23% under optimum conditions and 51–52% under drought stress for top-performing entries (Beyene et al. 2019). Therefore, the 161% superiority observed under drought in the present study substantially exceeds the the previous reports, while the 60.4% gain under optimum conditions was also notably higher than typical values **(Page 31 line 686-694 ).** |
| 17 | Expand the discussion on why the short ASI in Cluster 3 (1.02–1.35 days) is the "critical" physiological driver for drought resilience | The ASI ranged narrowly from 1.02 to 1.35 days across all clusters (Table 13), indicating strong synchrony between pollen shed and silk emergence among the evaluated hybrids. This consistently short ASI is physiologically important and widely recognized as a critical driver of drought resilience in maize. Under water stress, silk emergence is typically delayed more than anthesis, resulting in an extended ASI, reduced pollination success, and poor kernel set. In contrast, genotypes that maintain a short ASI, as observed in this study, preserve reproductive synchrony and ensure effective fertilization even under moisture-limited conditions (Bolafios and Edmeades 1996). Recent studies further confirm that ASI is a key indicator of stress sensitivity, where increased ASI reflects disrupted reproductive development and consequent yield reduction under drought stress (Cairns et al. 2013; Zhuang et al. 2024). This reinforces ASI as a robust physiological marker linking reproductive failure to yield loss under water-limited environments. The consistently short ASI identified in this study expressed alongside other favorable agronomic and physiological traits that contribute to the overall adaptation. This suggests that drought resilience in these hybrids is not governed by a single trait, rather by a coordinated suite of traits that collectively enhance reproductive stability and yield performance. (**page 32-33, line 719-731**) |
| 18 | Address how the 23.4% "unknown" background genotypes in Cluster 1 might contribute to the observed functional diversity. | This issue was addressed by obtaining additional clarification from the breeding program. Accordingly, out of the 133 lines grouped under unknown originally,112 were successfully re-distributed across the existing populations, while only 21 lines remained in an unknown group. As a result, the distribution of lines across clusters was only minimally affected, with no substantial change in the number of lines per cluster. Consequently, the overall interpretation remained consistent, with only negligible quantitative differences in cluster composition and magnitude. **(Table 10 and Table 12 on page 19 and 24, respectively and Fig 3 on page 21).** |
| 19 | Discuss why residual variance for ASI was 38% higher in well-watered conditions compared to stress. | **The discussion as follow:**  The residual variance for GY and ASI was higher under well-watered (optimum conditions), by 22 and 38%, respectively, as compared to under drought stress condition. The increase might be associated with the greater environmental heterogeneity in the combined optimum analysis. The optimum dataset included both Kakamega and Kiboko, which differ markedly in temperature, humidity, and evaporative demand. Such contrasts likely increased unexplained variability in ASI, a trait known to be highly sensitive to environmental fluctuations (Bolafios and Edmeades 1996; Cairns et al. 2013). In contrast, the managed drought stress at Kiboko provided a more uniform environment, reducing environmental noise and resulting in lower residual variance. This suggests that ASI is more environmentally plastic under heterogeneous optimum conditions than under controlled stress. **(Page 31, line 675 - 683)** |
|  |  |  |
|  |  |  |
|  | **Reviewer # 2** |  |
|  | **Abstract** |  |
| 1 | The abstract is overly long and could be reduced without loss of content. It is recommended to make it more concise, prioritizing objectives, methodological approach, key results, and conclusions, while avoiding excessive detail. | The comments are addressed |
|  | **Introduction** |  |
| 2 | The introduction contains an extensive review that could be shortened without compromising the study’s contextualization.  There is no clear need to address marker-assisted selection, as this topic is not developed throughout the manuscript.  . | The Introduction revised and shortened in response to the reviewer’s comments |
| 3 | Additionally, the abstract presents an explicit hypothesis; however, this hypothesis is neither revisited nor properly justified in the introduction. It is recommended that the authors clearly incorporate this hypothesis into the introduction, establishing a logical link between the scientific gap, study objectives, and experimental approach | The introduction section revised for such limitation**, page 3, line 88-95** |
|  | **Materials and Method** |  |
| 4 | The methodological description requires greater clarity and detail in several aspects:  The statement that “each of the 855 DH lines was crossed with a single-cross tester from the opposite heterotic group” introduces a conceptual inconsistency with the study hypothesis, which suggests the exploration of variability within the same genetic background. The use of a tester from an opposite heterotic group may introduce substantial genetic divergence and therefore requires clarification. The authors should explicitly explain this strategy and its relationship to the proposed hypothesis. | For clarity purpose, have revised the hypothesis as follows, while maintaining the methodological explanation as originally described for this comment;  We hypothesized that doubled haploid (DH) lines derived from biparental populations harbor sufficient within-population genetic variation to produce exploitable phenotypic diversity and adaptive differentiation, and that crossing each DH line with a single-cross tester from the opposite heterotic group provides an effective framework to capture this variation through hybrid performance. (**Abstract line 34-38, Page 3, Line 91 - 95)** |
| 5 | Table 1 lacks an adequate explanatory footnote, including full descriptions of abbreviations and presented information. This issue also applies to several other tables and figures. For example, what does “HG” refer to? | Foot note included for tables and figures: page 5 line 144-145, page 10 line 251, page 11 line 276-278, page 12 line 291-292, page 12 line 295-297, page 13 line 311-312, page 14 line 326-329, page 18 line 416, page 19 line 426-427, page 24 line 526-527, page 25 line 531-534, page 26 line 565-568, page 28 line 595-598, page 29 line 622-624 |
| 6 | The characterization of experimental environments is insufficient. Terms such as “well-watered” and “drought stress” require clear operational definitions. It is recommended to include:  irrigation management criteria; monitoring of drought stress; occurrence and amount of rainfall during the experimental period; indicators confirming that drought stress was effectively imposed. These details are essential for proper interpretation of the results. | We have tried to characterize the experimental environment as “drought stress was imposed by withholding irrigation approximately two weeks before flowering and maintaining water deficit conditions until harvest, allowing progressive soil moisture depletion. In contrast, well-watered trials received supplemental irrigation whenever visible signs of moisture stress were observed, ensuring near-optimal soil moisture throughout the growing period. Although the exact amount of water applied was not quantified in millimeters” **(page 4, line 115-120)** |
|  | **Discussion** |  |
| 7 | Multiple statements are not adequately supported by bibliographic references. For example, the claim of higher heritability under drought stress should be properly referenced. A careful revision is recommended to ensure consistent bibliographic support. | **We have tried to compare our findings with previous reports like:**  Narrow-sense heritability for GY, AD, PH, and EH was consistently higher under drought conditions. This indicates stronger additive genetic control in stress environments. The higher heritability observed under moisture stress at Kiboko suggests that genetic variance was more effectively expressed relative to environmental variance. As a result, genetic differences among genotypes became more detectable, improving selection efficiency.  Similar findings have been reported in tropical maize, where moderate-to-high heritability under managed drought enabled effective selection for grain yield and stress tolerance (Idehen, Abegunde, and Kehinde 2023; Sedhom et al. 2024a; Singamsetti et al. 2023; Tarekegne et al. 2023). These studies highlight that well-managed stress conditions can enhance the expression of additive genetic effects and accelerate genetic gain.  However, contrasting evidence also exists. Some studies report lower heritability under drought due to strong genotype × environment interaction and the complex genetic basis of stress tolerance (Alam et al. 2022; Amadu et al. 2025; Chen et al. 2023). Under severe or highly variable stress, environmental variance may dominate and reduce the expression of genetic potential. Similar trends were observed by (Elmyhun et al. 2024) and Beyene et al. (2013), where higher heritability was reported under optimal conditions due to more stable environments.  These differences indicate that heritability under drought is highly context-dependent. It is influenced by stress intensity, environmental uniformity, and population structure. In the present study, drought conditions at Kiboko were sufficiently discriminative to reveal additive genetic variation without masking phenotypic expression.  From a breeding perspective, these results emphasize the importance of selection under target stress environments. This approach improves the identification of genotypes with true drought tolerance. At the same time, multi-environment testing remains essential to ensure stable performance across diverse conditions. The CV for grain yield was highest under the Kiboko drought-stressed condition (37.73%), indicating increased variability under water-limited environments. This is consistent with previous studies in maize, where the coefficient of variation (CV) for grain yield is typically higher under drought stress than under well-watered conditions and can range from approximately 25.6% to 48.08% across different testing locations under water-deficit environments reported by Ningning et al. (2023). Such high variability reflects differential genotypic responses to stress and the reduction in mean yield under drought conditions. **(page 29-30, line 630- 656)** |
| 8 | The central hypothesis of the study is not revisited or discussed, which compromises the overall coherence of the manuscript. The discussion should be reorganized to explicitly address the proposed hypothesis | As per the suggestion, the manuscript has been revised and hypothesis of the study properly addressed under the discussion (Section 4.6.), **page 33-34, line 754- 784** |
| 9 | A relevant conceptual issue was not addressed: the apparent lack of substantial genetic distance among DH lines contrasted with the use of a tester from an opposite heterotic group. | We have tried to address it in the revised manuscript, page 34, line 774-776 |
| 10 | Considering that heterosis is associated with genetic divergence and heterotic group structure, this point should be explicitly discussed. | The point has been discussed in the revised manuscript, page 34, Line 776- 778. |
| 11 | The discussion is largely descriptive, lacking deeper interpretation and stronger integration with the literature. | The discussion section has been revised and appropriate reports have been cited to compare and support our findings |
|  | **Conclusion** |  |
| 12 | The conclusion largely repeats the results without effectively synthesizing the implications of the study.  There is no clear closure of the proposed hypothesis, indicating inconsistency between the introduction, development, and conclusion. The section should be reformulated to:  explicitly address the hypothesis;  highlight the scientific contributions;  indicate implications for plant breeding. | The comment is well taken and the conclusion section has been revised accordingly **(Page 35, line 803- 815)**. |

**One additional requirement**

**The request saying:** 1. In your Methods section, please provide additional information regarding the permits you obtained for the work. Please ensure you have included the full name of the authority that approved the field site access and, if no permits were required, a brief statement explaining why.

**Response:** We had already included this information in the Materials and Methods section of the main manuscript (page 4, lines 104–107), where we stated:

“This work did not require specific permission. One of the mandates of the International Maize and Wheat Improvement Center (CIMMYT) is to develop new lines and hybrids and evaluate them across different biotic and abiotic stresses to identify the best-adapted hybrids for commercialization across various parts of sub-Saharan Africa.”
